# Supratentorial and Infratentorial Lesions in Spinocerebellar Ataxia Type 3

**DOI:** 10.3389/fneur.2020.00124

**Published:** 2020-03-03

**Authors:** Po-Shan Wang, Yu-Te Wu, Tzu-Yun Wang, Hsiu-Mei Wu, Bing-Wen Soong, Chi-Wen Jao

**Affiliations:** ^1^Brain Research Center, National Yang-Ming University, Taipei, Taiwan; ^2^Institute of Biophotonics, National Yang-Ming University, Taipei, Taiwan; ^3^Department of Neurology, Taipei Municipal Gan-Dau Hospital, Taipei, Taiwan; ^4^Department of Biomedical Imaging and Radiological Sciences, National Yang-Ming University, Taipei, Taiwan; ^5^Department of Radiology, Taipei Veterans General Hospital, Taipei, Taiwan; ^6^Taipei Neuroscience Institute, Taipei Medical University, Taipei, Taiwan; ^7^Department of Neurology, Taipei Medical University-Shuang Ho Hospital, New Taipei City, Taiwan; ^8^Department of Neurology, Taipei Veterans General Hospital, Taipei, Taiwan; ^9^Department of Neurology, Shin-Kong Wu Ho Su Memorial Hospital, Taipei, Taiwan

**Keywords:** spinocerebellar ataxia type 3 (SCA3), supratentorial involvement, fractal dimension, morphological changes, MRI

## Abstract

**Background:** Spinocerebellar ataxia type 3 (SCA) is a cerebellum-dominant degenerative disorder that is characterized primarily by infratentorial damage, although less severe supratentorial involvement may contribute to the clinical manifestation. These impairments may result from the efferent loss of the cerebellar cortex and degeneration of the cerebral cortex.

**Method:** We used the three-dimensional fractal dimension (3D-FD) method to quantify the morphological changes in the supratentorial regions and assessed atrophy in the relatively focal regions in patients with SCA3. A total of 48 patients with SCA3 and 50 sex- and age-matched healthy individuals, as the control group, participated in this study. The 3D-FD method was proposed to distinguish 97 automatic anatomical label regions of gray matter (left cerebrum: 45, right cerebrum: 45, cerebellum: 7) between healthy individuals and patients with SCA3.

**Results:** Patients with SCA3 exhibited reduced brain complexity within both the traditional olivopontocerebellar atrophy (OPCA) pattern and specific supratentorial regions. The study results confirmed the extensive involvement of extracerebellar regions in SCA3. The atrophied regions of SCA3 in infratentorial and supratentorial cortex showed a wide range of overlapped areas as in two functional cortexes, namely cerebellum-related cortex and basal ganglia-related cortex.

**Conclusions:** Our results found that the atrophy of the SCA3 are not only limited in the infratentorial regions. Both cerebellar related cortex and basal ganglia related cortex were affected in the disease process of SCA3. Our findings might correlate to the common symptoms of SCA3, such as ataxia, Parkinsonism, dysarthria, and dysmetria. SCA3 should no longer be considered a disease limited to the cerebellum and its connections; rather, it should be considered a pathology affecting the whole brain.

## Introduction

Spinocerebellar ataxia type 3 (SCA3) is an inherited neurodegenerative disorder caused by CAG expansion in the coding region of chromosome 14q32.1 (1, 2). Clinically, patients with SCA3 exhibit cerebellar syndrome, Parkinsonism, ataxic gait, dysarthria, dysmetria, nystagmus, peripheral neuropathy, pyramidal, and extrapyramidal manifestations (3–5). Patients with SCA3 also suffered from emotional impairments, such as depression and anxiety (6, 7). These higher-order dysfunctions of SCA3 suggest the further involvement of extracerebellar structures rather than the conventional “olivopontocerebellar” pattern of neurodegeneration (8–12). Additionally, the SCA3 disorder has been described as cerebellar cognitive affective syndrome (CCAS) (6, 8).

Studies have used positron emission tomography (PET) and single-photon emission computed tomography to identify subclinical abnormalities in the cerebral cortex (13, 14). However, these studies reported the disruption of the cerebrocerebellar pathway cannot completely explain the cognitive and affective impairment in patients with SCA3. For example, visuospatial deficits in patients with SCA3 are markedly associated with the parietal lobe and are less connected to the cerebellum. Few studies have focused on the role of supratentorial regions in SCA3. The quantification of the degeneration in supratentorial regions may be crucial for evaluating dysfunction in patients with SCA3. In neuroimaging studies, measuring regional cortical atrophy is crucial for evaluating its association with cognitive impairment and emotional dysfunction.

Diffusion tensor imaging (DTI) can facilitate the visualization and characterization of white matter (WM), and DTI is also an efficient method for study of SCA3 (15). SCA3 had been verified as a WM dominant atrophy neurodegenerative disease (16). Previous studies showed patients with SCA3 demonstrate decreases in fractional anisotropy (FA) in the areas of cerebellum and brainstem, but increases in radial diffusivity (RD) in the cerebellum, brainstem, thalamus, and frontal and temporal lobes (17). Another DTI study identified widespread FA reduction in the bilateral cerebral-frontal, -parietal, -temporal, and -occipital WM; cerebellar WM; the thalamus; and the brainstem in patients with SCA3 (16). Moreover, mean diffusivity (MD) increases were detected in a similar, widely overlapping pattern in bilateral cerebral-frontal, -parietal, -temporal, and -occipital WM; cerebellar WM; the thalamus; and the brainstem (16).

In this study, we assessed atrophy in relatively focal regions and explained supratentorial involvement in SCA3. This study used the fractal dimension (FD) method, which has the advantage of producing results with minimal variation (18), to quantify cortical morphological changes and measure the regional cortical atrophy of supratentorial regions. In the present study, we measured the 3D-FD values of the 97 segments of the gray matter from the entire brain for each participant. We further compare these gray matter lesions results with DTI analysis of white matter lesions in SCA3 of our recent study. We anticipated the assessed GM lesions regions in this study are related to WM lesions regions from DTI study, and the result may verify the association between motor-related impairment and supratentorial regions atrophy in patients with SCA3.

## Materials and Methods

### Participants

A total of 48 patients with SCA3 and 50 sex- and age-matched healthy individuals, as the control group, participated in this study. This study was conducted in accordance with the Declaration of Helsinki and was approved by the Institutional Review Board of Taipei Veterans General Hospital. All participants provided written informed consent before participating in this study. All participants were recruited from the Department of Radiology, Taipei Veterans General Hospital. The SARA (scale for the assessment and rating of ataxia) was applied for clinical assessment of ataxia of patients with SCA3 (19). The symptoms of Parkinson's disease progress in SCA3 were measured by using Modified Hoehn and Yahr staging (20). The SARA score, which was a rating of the severity of ataxia symptoms ranging from 0 to 40, was used as a reference to indicate the progression of clinical severity in comparison with the cerebellar degeneration. A self-reported age of onset, the age at which the patients showed the first sign of any ataxic symptom, was acquired from each patient (21). The CAG repeat length of each SCA3 patient was determined by polymerase chain reaction, as described previously (22). Table 1 presents the demographic, clinical, and MR imaging data of both groups. Patients with SCA3 met the inclusion criteria if they had progressive and otherwise unexplained ataxia and tested positive for the SCA3 genotype. The disease duration in patients with SCA3 was 8.89 ± 6.432 years. Those in the control group had no central nervous system disease and did not exhibit any neurological abnormalities during the study period. An experienced neuroradiologist examined the T1- and T2-weighted images of the control group to ensure the absence of uncovered signs of another neurological disease or unexpected abnormalities.

**Table 1:** Demographic, clinical, and MR image data of the control group and the patient group.

**Characteristic**	**Group**		
	**Controls (*****N*** **=** **50)**	**SCA3 Patients (*****N*** **=** **48)**	*****p***-value**
Sex (F/M)	25/25	21/27	0.535^a^
Age (years)^†^	48.24 ± 13.956	48.13 ± 11.747	0.516^b^
Duration (years)^†^	–	8.89 ± 6.432	–
SARA^†^	–	14 ± 8.103	–
H & Y staging^†^	–	2.88 ± 1.19	–
Cerebral atrophy/cerebellar atrophy observed through visual inspection	–	9/39	–
CAG repeat length	–	73.2 ± 4.2 CAG < 74/CAG > 74 = 26/22	–

*SARA, Scale for the Assessment and Rating of Ataxia; MMSE, Mini-Mental State Examination; H & Y staging, Hoehn and Yahr Staging Scale*.

a *Pearson's chi-square test (χ^2^ = 0.384)*;

b *2-tailed 2-sample t-test*.

† *Continuous variables are expressed as mean ± standard deviation*.

### Magnetic Resonance Imaging

#### Data Acquisition and Processing

**Figure 1:**
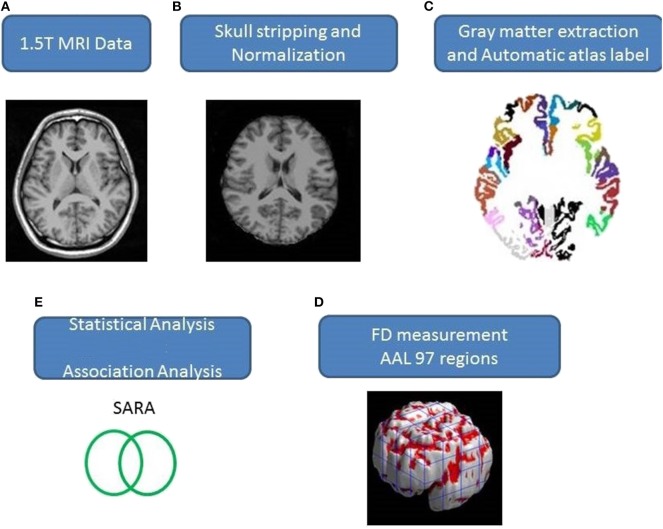
**(A–E)** Procedure of the image process and statistical analysis.

Axial MR human brain images covering the entire cerebrum and cerebellum were acquired using a 1.5-T Vision Siemens scanner (Erlangen, Germany). Participants were scanned with a circularly polarized head coil to obtain T1-weighted images (TR = 14.4 ms; TE = 5.5 ms; matrix size: 256 × 256; 1.5 mm axial slices; FOV = 256 × 256 mm^2^; voxel size, 1.0 × 1.0 × 1.5 mm^3^, number of slice = 128). The acquired T1-weighted images of each participant were reformatted into an axial image and converted to an analysis format by MRIcro software (Chris Rorden, University of Nottingham, UK; www.sph.sc.edu/comd/rorden/mricro.html). Figure 1 illustrates the data processing and statistical analysis flowchart. To improve the accuracy of brain tissue extraction, an automated skull-stripping function was applied to image volumes using the brain extraction tool in MRIcro software (Figure 1B). The subsequent processes were performed using DiffeoMap (Li, X.; Jiang, H.; and Mori, S.; Johns Hopkins University, www.MriStudio.org). In this procedure, a 12-parameter affine transformation (23) was used to normalize each T1-weighted image toward the JHU_MNI_SS_T_ss T1 template.

#### Atlas Extraction

The image data processes were performed using the SPM8 toolbox (Wellcome Department of Cognitive Neurology, Institute of Neurology, University College London, London, UK, http://www.fil.ion.ucl.ac.uk/spm/) and the IBASPM toolbox (Individual Brain Atlases using Statistical Parametric Mapping, http://www.thomaskoenig.ch/Lester/ibaspm.htm), both run using MATLAB 2010 software (Mathworks, Natick, MA, USA).

The procedure involves three steps after normalization: (1) segmentation of the normalized image into gray matter, white matter, and cerebral spinal fluid in native space, (2) parcellation of gray matter into 116 regions based on the anatomical labeling of the Montreal Neurological Institute (MNI) anatomical atlas (24), and (3) transformation of gray matter images into the MNI space and the anatomical alignment of each voxel of gray matter to the 116 automatic anatomical label structures using IBASPM (24), left cerebrum: 45 regions, right cerebrum: 45 regions, cerebellum: 26 regions; (Figure 1C). The 26 regions of the cerebellum were merged into seven regions according to their anatomical structures, and the volumes of the 97 labeled (45 for each cerebral hemisphere and seven for cerebellum, Supplementary Table 1) brain structures were extracted for each person (Figure 1C).

The FD method was originally proposed by Mandelbrot for the quantification of the shape-related complexity of objects into a single numerical value (25). The FD method is used for the topological measurement of complexity; a higher FD value represents greater topological complexity of the tissue under study (18, 26). Many neurologists have demonstrated that the FD value can serve as a quantitative measure for accurately describing the morphological complexity of the cerebral folding (18, 26, 27). Because FD analysis is based on a logarithmic scale, even a small increase in the FD value may correspond to a considerable increase in complexity (18). This study adopted the 3D box-counting method to measure the 3D-FD values of the 97 segments of gray matter from the entire brain for each participant (Figure 1D). A related study details the algorithm for the box-counting method (18).

### Statistical Analysis

The sex- and age-related differences between the groups were measured using Pearson's chi-square test (χ^2^ = 0.384, *p* = 0.535) and the 2-tailed 2-sample *t*-test (*p* = 0.516), separately. Linear regression was applied to remove effects of age and gender. We adopted a 2-tailed *t*-test to determine whether a significant difference existed between the control and patient groups regarding the 3D-FD values of each cortical region. The significance of the results was based on the false discovery rate (FDR-corrected *p* = 0.05) (28). The magnitude of the association between the 3D-FD value of individual brain regions and clinical features, such as disease duration and SARA, was determined through Pearson's r measurement (Figure 1E). These analyses were conducted using the Statistics Toolbox in MATLAB 2010.

## Results

### Patients With SCA3 Revealed Significant Lesions in Typical Infratentorial Lesions Regions

**Figure 2:**
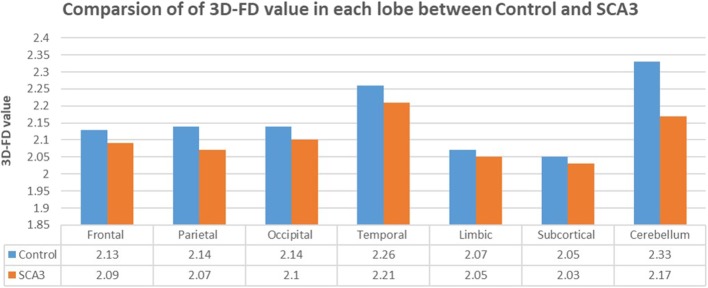
Comparison of 3D-FD values in each lobe between the healthy group and the SCA3 group. The 3D-FD value in each lobe of SCA3 showed significantly lower the control group. The *p*-value of significant difference of cerebellum, frontal lobe, parietal lobe, and occipital lobe are smaller than 0.01, and for other lobes are smaller than 0.05.

Experienced neuroradiologists reported the MRI findings for patients with SCA3 (Table 1). Figure 2 illustrates the comparison of 3D-FD values in each lobe between the healthy group and the SCA3 group. Globally, patients with SCA3 exhibited significantly decreased 3D-FD values in every lobe, and the cerebellum was associated with the most substantial reduction in 3D-FD value (Figure 2). The 3D-FD values of the cerebellar cortex of SCA3 showed significant correlation with their SARA scores (*r* = −0.3346; *p* = 0.023). We further parcellated the cerebral and cerebellar cortex into 97 regions and quantified atrophy in each parcellated region. Patients with SCA3 had typical infratentorial lesions regions, including pontine nuclei, cerebellar cortex, and inferior olives. The details of the significantly decreased 3D-FD values of atrophy regions between the healthy group and the SCA3 group are summarized in Table 2.

**Table 2:** Significant atrophied regions in patients with SCA3 (*p* < 0.05).

**Region (L/R)**	**Controls**	**SCA3**
**Cerebellar cortex**		
Entire	2.56 ± 0.02	2.53 ± 0.04
Anterior lobe (L)	2.17 ± 0.04	2.11 ± 0.07
Anterior lobe (R)	2.15 ± 0.04	2.03 ± 0.08
Posterior lobe (L)	2.47 ± 0.03	2.45 ± 0.04
Posterior lobe (R)	2.48 ± 0.03	2.44 ± 0.04
Vermis	2.15 ± 0.05	2.12 ± 0.04
**Frontal lobe**		
Precentral gyrus (L)	2.15 ± 0.07	2.07 ± 0.07
Superior frontal gyrus (L)	2.08 ± 0.03	2.05 ± 0.05
Superior frontal gyrus (R)	2.13 ± 0.04	2.10 ± 0.06
Middle frontal gyrus (L)	2.28 ± 0.04	2.25 ± 0.04
Orbitofrontal cortex (superior-medial) (L)	2.11 ± 0.04	2.08 ± 0.05
Orbitofrontal cortex (superior-medial) (R)	2.14 ± 0.04	2.10 ± 0.05
Inferior frontal gyrus (opercular) (R)	2.10 ± 0.05	2.07 ± 0.05
Inferior frontal gyrus (triangular) (L)	2.27 ± 0.04	2.23 ± 0.05
Supplementary motor area (L)	2.19 ± 0.05	2.14 ± 0.05
Superior frontal gyrus (medial) (L)	2.17 ± 0.04	2.12 ± 0.06
Superior frontal gyrus (medial) (R)	2.09 ± 0.05	2.06 ± 0.07
Paracentral lobule (L)	2.04 ± 0.07	1.99 ± 0.08
Paracentral lobule (R)	1.98 ± 0.07	1.92 ± 0.08
**Parietal lobe**		
Post-central gyrus (L)	2.17 ± 0.05	2.10 ± 0.06
Superior parietal gyrus (L)	2.10 ± 0.06	2.03 ± 0.06
Superior parietal gyrus (R)	2.08 ± 0.06	2.04 ± 0.07
Inferior parietal gyrus (L)	2.19 ± 0.07	2.09 ± 0.08
Supramarginal gyrus (L)	2.11 ± 0.05	2.04 ± 0.06
Angular gyrus (L)	2.12 ± 0.07	2.00 ± 0.09
Precuneus (L)	2.21 ± 0.03	2.17 ± 0.05
Precuneus (R)	2.14 ± 0.04	2.10 ± 0.04
**Temporal lobe**		
Superior temporal gyrus (L)	2.181 ± 0.051	2.120 ± 0.053
Middle temporal gyrus (L)	2.336 ± 0.034	2.303 ± 0.048
**Occipital lobe**		
Calcarine fissure and surrounding cortex (L)	2.25 ± 0.04	2.22 ± 0.04
Cuneus (L)	2.13 ± 0.04	2.11 ± 0.04
Lingual gyrus (L)	2.20 ± 0.04	2.17 ± 0.05
Superior occipital gyrus (L)	1.95 ± 0.06	1.89 ± 0.08
Middle occipital gyrus (L)	2.19 ± 0.05	2.12 ± 0.07
Temporal lobe		
Superior temporal gyrus (L)	2.18 ± 0.05	2.12 ± 0.05
Middle temporal gyrus (L)	2.34 ± 0.03	2.30 ± 0.05
**Limbic**		
Posterior cingulate gyrus (L)	1.99 ± 0.05	1.95 ± 0.05
Parahippocampal gyrus (R)	2.16 ± 0.03	2.14 ± 0.03
**Subcortical regions**		
Caudate nucleus (R)	2.09 ± 0.04	2.04 ± 0.05
Lenticular nucleus, putamen (L)	2.11 ± 0.08	2.07 ± 0.05
Amygdala (R)	1.94 ± 0.04	1.97 ± 0.04
Caudate nucleus (L)	2.08 ± 0.05	2.05 ± 0.05

*Brain regions with significant difference (P < 0.01) in 3D-FD values between control and patients with SCA3. Significant difference under a corrected threshold of FDR = 0.05, The 3D-FD values are expressed as mean ± standard deviation; L, left; R, right*.

### Patients With SCA3 Revealed Cerebral Lateralized Supratentorial Lesions

**Figure 3:**
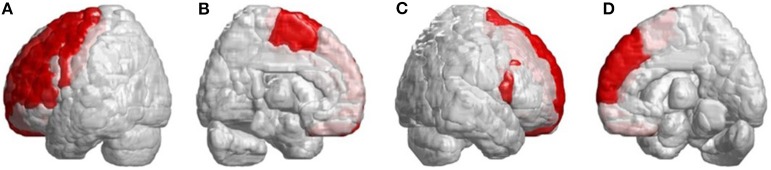
Regions within the frontal lobe indicating lower 3D-FD values in patients with SCA3 compared with controls. **(A)** Left-outer view, **(B)** left-inner view, **(C)** right-outer view, and **(D)** right-inner view. Patients with SCA3had significantly decreased 3D-FD values in frontal regions, including the premotor cortex (left precentral gyrus, bilateral superior frontal gyrus, and left-middle frontal gyrus), supplementary motor cortex (left supplementary motor area), and primary motor cortex (left precentral gyrus). Other atrophied regions were observed in the left inferior frontal gyrus (opercular), left inferior frontal gyrus (triangular), bilateral orbitofrontal cortex (superior-1 medial), bilateral superior frontal gyrus (medial), and bilateral paracentral lobule in patients with SCA3. All the significantly decreased 3D-FD values regions are illustrated in red.

Beside the infratentorial lesions, lesions in supratentorial regions were also observed in all patients with SCA3. They showed widespread lesions in 39 cerebral parcellated regions (*p* < 0.05), including frontal lobe, parietal lobe, occipital lobe, and temporal lobe. They revealed lateralized atrophy, and predominantly in the left hemisphere (right/left: 12/27). Especially in the occipital and temporal lobes, patients with SCA3 revealed significantly decreased 3D-FD values only in the left hemisphere. The significant atrophied regions in frontal regions were the premotor cortex (left precentral gyrus, bilateral superior frontal gyrus, and left-middle frontal gyrus), supplementary motor cortex (left supplementary motor area), and primary motor cortex (left precentral gyrus) (Figure 3).

**Figure 4:**
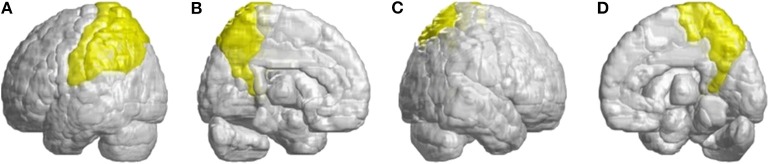
Regions within the parietal lobe indicating a decrease in 3D-FD value in patients with SCA3 compared with controls. **(A)** Left-outer view, **(B)** left-inner view, **(C)** right-outer view, and **(D)** right-inner view. Compared with controls, patients with SCA3had significantly lower 3D-FD values in the left postcentral gyrus, left supramarginal gyrus, left angular gyrus, bilateral superior parietal gyrus, and bilateral precuneus of the parietal lobe. All the significantly decreased 3D-FD values regions are illustrated in yellow.

Other atrophied regions were in the left inferior frontal gyrus (opercular), left inferior frontal gyrus (triangular), bilateral orbitofrontal cortex (superior-medial), bilateral superior frontal gyrus (medial), and bilateral paracentral lobule. In parietal lobe, decreased FD values were detected in the left postcentral gyrus, left supramarginal gyrus, left angular gyrus, bilateral superior parietal gyrus, and bilateral precuneus of patients with SCA3 (Figure 4).

**Figure 5:**
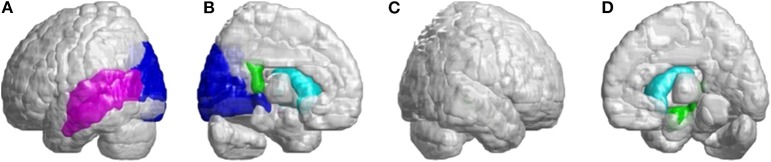
Regions within the occipital lobe, temporal lobe, and subcortical lobe indicating significantly lower 3D-FD values in patients with SCA3 compared with controls. **(A)** Left-outer view, **(B)** left-inner view, **(C)** right-outer view, and **(D)** right-inner view. The blue regions show significantly lower 3D-FD values in the occipital lobe, including the calcarine fissure and the surrounding cortex, cuneus, and lingual gyrus and the superior and middle occipital gyrus in the left hemisphere. Purple regions denote lower 3D-FD values in the left-superior temporal gyrus and left-middle temporal gyrus. Aqua blue regions represent lower 3D-FD values in subcortical regions, including the bilateral caudate and left putamen. Green regions represent significantly lower 3D-FD values in the left-posterior cingulate gyrus and parahippocampal gyrus of the limbic system.

In the occipital lobe, SCA3 patients revealed significant lower FD values in the calcarine fissure and the surrounding cortex, cuneus, lingual gyrus, and the superior and middle occipital gyri in the left hemisphere (Figure 5A, blue color). In the temporal lobe, regions with decreased FD values were in the left-superior temporal and left-middle temporal gyri of patients with SCA3 (Figure 5A, purple color). The 3D-FD values in the left-posterior cingulate gyrus of the limbic region were also significantly decreased in patients with SCA3 (Figures 5B,D, green color). Regions of significant atrophy were observed in the basal ganglia, including the bilateral caudate nucleus and left putamen, in patients with SCA3 (Figures 5B,D, aqua blue color). In the right-outer occipital lobe, no significant atrophied regions were observed in patients with SCA3 (Figure 5C).

**Figure 6:**
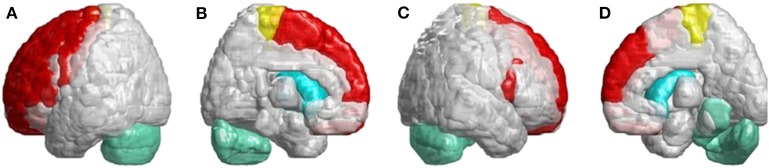
SCA3 is involved in both the cerebellar–thalamocortical and basal ganglia–thalamocortical pathways. **(A)** Left-outer view, **(B)** left-inner view, **(C)** right-outer view, and **(D)** right-inner view. In the cerebellar–thalamocortical loop, patients with SCA3 exhibit significantly lower 3D-FD values in the cerebellar, premotor, and supplementary motor cortexes. The decreased 3D-FD values in the cerebellar cortex and other motor-related cerebral regions in patients with SCA3 provide an explanation for the presence of clinical symptoms. In the basal ganglia–thalamocortical loop, patients with SCA3 exhibit significantly lower 3D-FD values in the frontal cortex, primary motor cortex, putamen, and caudate. These results reveal morphological changes in the basal ganglia–thalamocortical loop in patients with SCA3.

In Figure 6, we found many cerebral atrophied regions included cerebellum, cerebellar–thalamocortical, and basal ganglia–thalamocortical link circuits that implied patients with SCA3 revealed cerebellar–thalamocortical and basal ganglia–thalamocortical damaged in these pathways.

## Discussion

Clinically, patients with SCA3 exhibit cerebellar syndrome and parkinsonism (3–5). The atrophied regions of infratentorial and supratentorial cortex listed in Table 2, and showed a wide range of overlapped areas as in two functional regions, namely cerebellum- related (CB-related) cortex (29) and basal ganglia-related (BG-related) cortex (30). The CB-related cortex include the cerebellum, prefrontal, sensorimotor cortices, prefrontal cortex, and temporal lobe, and BG-related cortex include primary motor cortex, supplementary motor area, premotor cortex, and basal ganglia. Our results revealed consistent with previous conventional neuroimaging studies that the cerebellum is the most affected and atrophied region in patients with SCA3 (4, 31, 32). Additionally, the 3D-FD value in the cerebellar cortex of patients with SCA3 had a significantly negative correlation with SARA scores (*r* = −0.3346; *p* = 0.023) as Rezende et al. had ever reported (33).

In the infratentorial regions, patients with SCA3 had a significant decreased 3D-FD values in putamen and caudate. Neuropathological studies have shown neuronal loss in the putamen and caudate, which may lead to basal ganglia atrophy in association with Parkinsonism (34). Parkinsonian features are the prevalent phenotype of the SCA3 mutation and are usually accompanied by basal ganglia symptoms (35–37). In necropsy studies, the involvement of the basal ganglia is common in patients with SCA3. Functional imaging using PET studies has also demonstrated early functional decline of the caudate and putamen in patients with SCA3. Additionally, patients with SCA3 had a significantly smaller basal ganglia volume (35). However, related studies have focused only on the basal ganglia and infratentorial regions. In Figure 6, we verified that SCA3 may not only have the basal ganglia lesions but also the cerebellar–thalamocortical and basal ganglia–thalamocortical pathways damage.

Patients with SCA3 exhibit significantly decreased 3D-FD values of the premotor cortex and supplementary motor cortex in their cerebellar–thalamocortical loop. The premotor cortex and supplementary motor area are associated with movement control, such as muscle force and direction of reach. Damage in these regions may induce ataxia of gait, stance, and limb, in addition to movement decomposition (38, 39). The reduced 3D-FD values of cerebellar cortex and other motor-related cerebral regions in patients with SCA3 may explain the presence of clinical symptoms. Additionally, in the basal ganglia–thalamocortical loop, patients with SCA3 exhibit significantly decreased 3D-FD values in the frontal and primary motor cortexes. The primary motor cortex is also a major destination for basal ganglia output (40). Disturbances of the basal ganglia–thalamocortical loop may also contribute to Parkinsonian motor dysfunction (41). The results of this study emphasize the morphological changes in the basal ganglia–thalamocortical loop in patients with SCA3, which previous neuroimaging studies have failed to address.

Recently, we used diffusion tensor imaging (DTI) to measure water molecular diffusion of white matter (WM) alteration in SCA3 (15). We found SCA3 revealed widespread white matter lesions in the bilateral cerebral-frontal, -parietal, -temporal, and -occipital WM; cerebellar WM; the thalamus, and the brainstem. Identically, in this study we observed the SCA3 showed almost in the same parcellated regions of these WM lesions that revealed GM lesions. These current findings confirm the involvement of supratentorial regions in patients with SCA3. Braga et al. reported similar findings, demonstrating that SCA3 causes CCAS through the impairment of executive functions, verbal fluency, abstract reasoning, and working memory (8). In neuropathological studies, it was revealed that CCAS results from the disruption of the cerebellar circuit, which is associated with the prefrontal, superior parietal, superior temporal, and limbic cortexes (42, 43). Studies have confirmed the role of the cerebellum in cognitive function; however, cognitive, affective, and visuospatial functions are directly associated with cortical degeneration. The significantly atrophied areas of the supratentorial regions in patients with SCA3 were more extensive than the CCAS-associated regions. The atrophy of the supratentorial cortex may be the primary factor associated with the dysfunction of the ataxia and Parkinsonism-related regions. These findings suggest that SCA3 should no longer be considered a disease limited to the cerebellum and its connections; rather, it should be considered a pathology affecting the whole brain.

However, this study still had certain limitations. The main limitations in this study are the lack of specific cognitive evaluations and clinical parameters for assessing CCAS in SCA. Further investigations are warranted regarding the correlation between the present results and clinical data, such as functional MRI data. This may help provide a more precise method for assessing the association between cognitive with motor-related involvement and regional atrophy in patients with SCA3.

## Conclusions

In this study, we used the 3D-FD method to quantify regional morphological variations in patients with SCA3 and assess the association between supratentorial atrophy and motor-related involvement in patients with SCA3. Our results found significant degeneration of the cerebral cortex in specific regions, which maybe cerebellar related or basal ganglia related. Our findings might correlate to the common symptoms of SCA3, such as ataxia, Parkinsonism, dysarthria, and dysmetria. SCA3 should no longer be considered a disease limited to the cerebellum and its connections; rather, it should be considered a pathology affecting the whole brain.

## Data Availability Statement

All datasets generated for this study are included in the article/Supplementary Material.

## Ethics Statement

The studies involving human participants were reviewed and approved by Institutional Review Board of Taipei Veterans General Hospital. The patients/participants provided their written informed consent to participate in this study.

## Author Contributions

P-SW and Y-TW organized the research project, reviewed and critiqued the manuscript. T-YW wrote manuscript. H-MW accessed research data. B-WS organized the research and performed data collection. C-WJ performed the statistical analysis, wrote, and revised the manuscript.

### Conflict of Interest

The authors declare that the research was conducted in the absence of any commercial or financial relationships that could be construed as a potential conflict of interest.
