# Supplementary material for: Supratentorial and Infratentorial Lesions in Spinocerebellar Ataxia Type 3
Source: Front Neurol. 2020 Mar 3;11:124. doi: 10.3389/fneur.2020.00124 (PMC7062793; doi:10.3389/fneur.2020.00124)
Supplement: Supplementary file 1 [file Data_Sheet_1.PDF]

AAL97 Parcellation table

lobe	Index		Regions	abbreviations
	left	right		
Frontal lobe	1	2	Precentral gyrus	PreC
	3	4	Superior frontal gyrus, dorsolateral	SFGdor
	5	6	Superior frontal gyrus, orbital	ORBsup
	7	8	Middle frontal gyrus	MFG
	9	10	Middle frontal gyrus, orbital	ORBmid
	11	12	Inferior frontal gyrus, opercular	IFGoper
	13	14	Inferior frontal gyrus, triangular	IFGtraing
	15	16	Inferior frontal gyrus, orbital	ORBinf
	17	18	Rolandic operculum	ROL
	19	20	Supplementary motor area	SMA
	21	22	Olfactory cortex	OLF
	23	24	Superior frontal gyrus, medial	SFGmed
	25	26	Superior frontal gyrus, medial orbital	ORBsupmed
	27	28	Gyrus rectus	REC
Limbic lobe	29	30	Insula	INS
	31	32	Anterior cingulate gyrus	ACC
	33	34	Median- and para-cingulate gyrus	MCC
	35	36	Posterior cingulate gyrus	PCC
	37	38	Hippocampus	HIP
	39	40	supramarginal gyrus	PHIP
	41	42	Amygdala	AMY
Occipital lobe	43	44	Calcarine fissure and surrounding cortex	CAL
	45	46	Cuneus	CUN
	47	48	Lingual gyrus	LIN
	49	50	Superior occipital gyrus	SOG
	51	52	Middle occipital gyrus	MOG
	53	54	Inferior occipital gyrus	IOG
	55	56	Fusiform gyrus	FUS

**Parietal lobe**	57	58	Postcentral gyrus	PostC
	59	60	Superior parietal gyrus	SPG
	61	62	Inferior parietal gyrus	IPG
	63	64	Supramarginal gyrus	SM
	65	66	Angular gyrus	ANG
	67	68	Precuneus	PCUN
	69	70	Paracentral lobule	PL
**Basal ganglia**	71	72	Caudate nucleus	CAU
	73	74	Lenticular nucleus, putamen	PUT
	75	76	Lenticular nucleus, pallidum	PAL
	77	78	Thalamus	THA
**Temporal lobe**	79	80	Heschl gyrus	HES
	81	82	Superior temporal gyrus	STG
	83	84	Superior temporal gyrus, temporal pole	TPOsup
	85	86	Middle temporal gyrus	MTG
	87	88	Middle temporal gyrus, temporal pole	TPOmid
	89	90	Inferior temporal gyrus	ITG
**Cerebellum**	91	92	Cerebelum anterior lobe	CbAL
	93	94	Cerebelum posterior lobe upper	CbPLU
	95	96	Cerebelum posterior lobe lower	CbPLL
	97		Cerebelum Vermis	Vermis
